# Pre-IVM with C-type natriuretic peptide promotes mitochondrial biogenesis of bovine oocytes via activation of CREB

**DOI:** 10.1038/s41598-024-67094-7

**Published:** 2024-07-15

**Authors:** Zehua Zhang, Zhenwei Jia

**Affiliations:** College of Animal Science and Technology, Inner Mongolia Minzu University, 536 West Huolinhe Street, Tongliao, 028000 Inner Mongolia People’s Republic of China

**Keywords:** CNP, Bovine oocyte, Mitochondrial biogenesis, CREB, Cell biology, Developmental biology

## Abstract

The aim of this study was to evaluate the effects of C-type natriuretic peptide (CNP) treatment prior to in vitro maturation (IVM) on mitochondria biogenesis in bovine oocyte matured in vitro and explore the related causes. The results showed that treatment with CNP before IVM significantly improved mitochondrial content, elevated the expression of genes related to mitochondria biogenesis, and increased the protein levels of phosphorylation of cAMP-response element binding protein (p-CREB) in bovine oocytes following IVM. However, further studies revealed that treatment with CNP before IVM could not increased the protein levels of p-CREB in bovine oocytes when natriuretic peptide receptor 2 activities was inhibited using the relative specific inhibitor Gö6976. In addition, treatment with CNP before IVM could not improved mitochondrial content or elevated the expression of genes related to mitochondria biogenesis in bovine oocytes when CREB activities was abolished using the specific inhibitor 666–15. In summary, these results provide evidence that treatment of bovine oocytes with CNP before IVM promotes mitochondrial biogenesis in vitro, possibly by activating CREB.

## Introduction

It has been well known that C-type natriuretic peptide (CNP) plays a key role in sustaining oocyte meiotic arrest^1–3^. In addition to its role in sustaining meiotic arrest, some studies have showed that CNP treatment for up to 6 h prior to in vitro maturation (IVM) enhanced the developmental competence of bovine oocytes maturated in vitro, implying that CNP has favorable effects on oocyte cytoplasmic maturation^3–5^. Mitochondria are the most prominent organelles in oocytes, which participate in diverse biological processes including ATP synthesis, calcium homeostasis, apoptosis and signal transduction^6,7^. In particular, mitochondria biogenesis plays a central role in mitochondrial functions, which is an important determinant of oocyte cytoplasmic maturation^8,9^.

Mitochondrial biogenesis is a complex process that occurs as a result of activation of several transcription factors in response to diverse biological stimuli. Among them, peroxisome proliferator-activated receptor co-activator 1α (PGC-1α) is known to be a central transcriptional coactivator that induces mitochondrial biogenesis by regulating the gene expression of nuclear respiratory factor (NRF1 and NRF2) and transcription factor A mitochondrial (TFAM), thus resulting in the upregulation of mitochondrial proteins encoded by both nuclear and mitochondrial genomes^10,11^. Notably, a previous study has shown that increased levels of cyclic guanosine 3′,5′-monophosphate (cGMP) induced by brain natriuretic peptide in human skeletal muscle cells increased the mRNA expression of genes involved in mitochondrial biogenesis^12^. Interestingly, CNP has been indicated to maintain higher levels of cGMP and adenosine 3′,5′-monophosphate (cAMP) and cyclic guanosine 3′,5′-monophosphate (cGMP) in oocytes by activating the receptor natriuretic peptide receptor 2 (NPR2) in cumulus cells^1,13^. More importantly, it has been reported that CNP treatment before IVM increased mtDNA copy number in bovine oocytes following IVM^4^. These results indicate that CNP pre-IVM may enhance mitochondrial biogenesis in bovine oocyte matured in vitro.

Notably, cAMP-response element binding protein (CREB) has been regarded as an important activator of PGC-1 α transcription to regulate mitochondrial biogenesis^14^. Importantly, there are studies showing that increased levels of cGMP and cAMP can enhance CREB activity^15,16^. This led us to hypothesize that in addition to inhibiting meiotic maturation of bovine oocytes, CNP pre-IVM treatment may lead to enhanced mitochondria biogenesis via the activation of CREB. Therefore, we investigated the effect of CNP pre-IVM treatment on mitochondria content, gene expression associated with mitochondrial biogenesis and protein levels of p-CREB in bovine oocytes maturated in vitro, then evaluated whether inhibition of CREB activity prior to IVM can abrogate the beneficial effect of CNP on mitochondria biogenesis in bovine oocytes after IVM. Meanwhile, we also evaluated whether the effects of CNP on CREB activity are related to NPR2 activation. The current results may provide insights into the mechanism underlying enhancement of mitochondrial biogenesis by treating bovine oocyte with CNP before IVM.

## Results

### CNP treatment before IVM promotes mitochondrial biogenesis in oocytes matured in vitro

**Figure 1:**
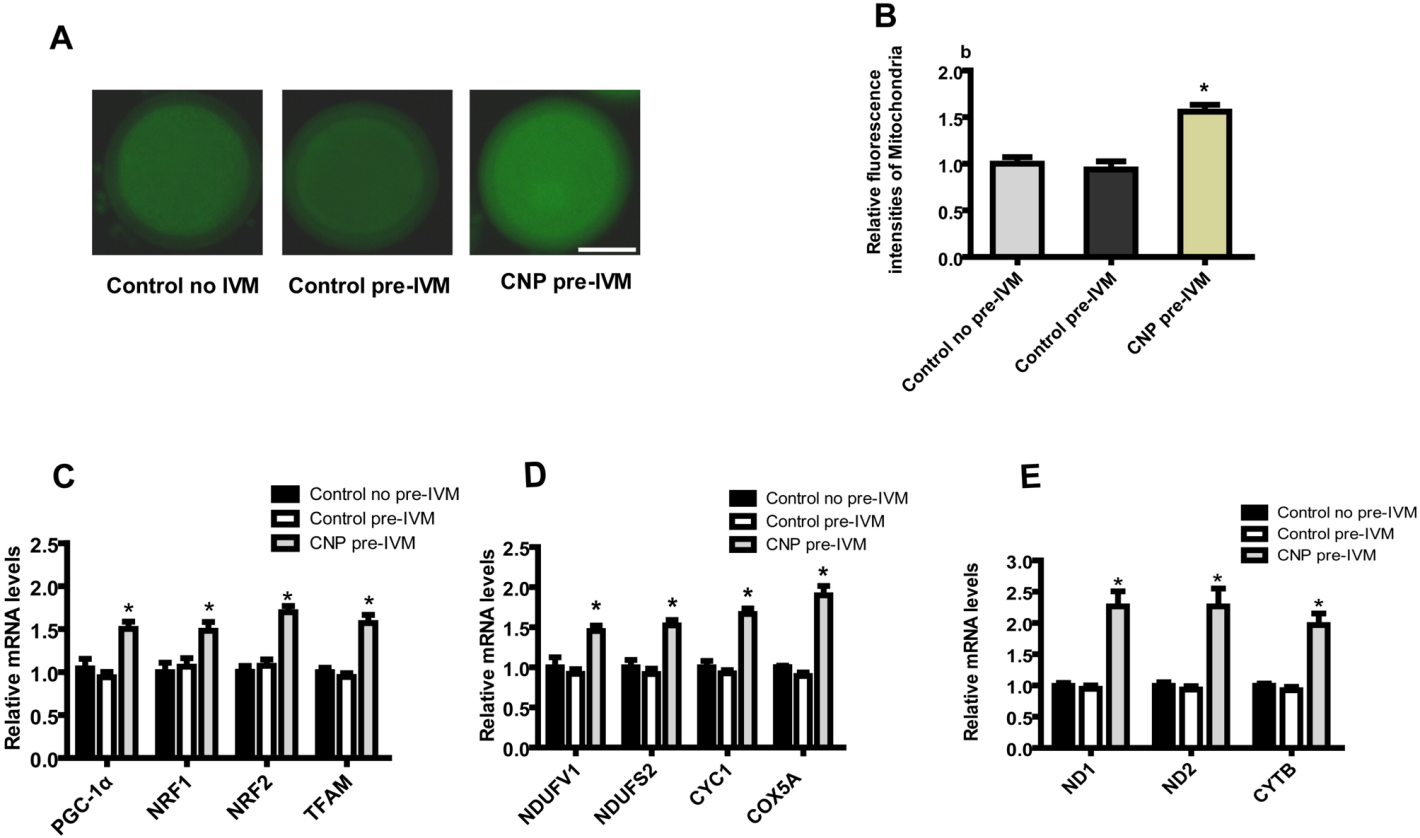
Effects of CNP treatment before IVM on mitochondria biogenesis during bovine oocytes maturation. Immature cumulus-oocyte complexes (COCs) were subjected to IVM for 24 h (Control no pre-IVM), or cultured in pre-IVM medium supplemented without or with CNP (Control pre-IVM and CNP pre-IVM, respectively) for 6 h, followed by 24 h of IVM. (**A**) Representative images of the oocyte stained with MitoTracker green in each treatment group after IVM. Scale bar = 50 µm. (**B**) Relative fluorescence intensities of the mitochondrial content in each treatment group oocytes after IVM (56 oocytes/group). (**C**–**E**) Relative mRNA transcript abundance of genes modulating mitochondria biogenesis, mitochondrial proteins genes encoded by both nuclear and mitochondrial genomes in bovine oocytes from each treatment group after IVM (150 oocytes/per group). Compared with the oocytes in the control (no pre-IVM) and pre-IVM (no CNP) group, *P < 0.05. The data are from at least three independent experiments.

To examine the effect of CNP pre-IVM treatment on mitochondrial biogenesis in bovine oocytes, collected immature cumulus-oocyte complexes (COCs) were either directly in vitro matured for 24 h (Control no pre-IVM), or cultured in basic pre-IVM medium supplemented without or with CNP (Control pre-IVM and CNP pre-IVM, respectively) for 6 h, followed by IVM for 24 h. After IVM, intra-oocyte mitochondrial intensity, and the expression levels of genes related to mitochondrial biogenesis in oocytes of each treatment group were tested. As shown in Fig. 1, the quantification of fluorescence intensity showed that the mitochondria content was significantly higher in the CNP pre-treated oocytes compared with the control and pre-IVM (no CNP) oocytes after IVM (*P* < 0.05; Fig. 1A,B). In addition, the gene expression of key regulators of mitochondria biogenesis, nuclear and mitochondrial genome encoding the respiratory chain complexes in CNP pre-IVM treatment group were significantly higher than that in the control (no pre-IVM) and pre-IVM (no CNP) group after IVM (*P* < 0.05; Fig. 1C,D,E).

### CNP treatment before IVM increases the protein levels of p-CEEB in oocytes matured in vitro

**Figure 2:**
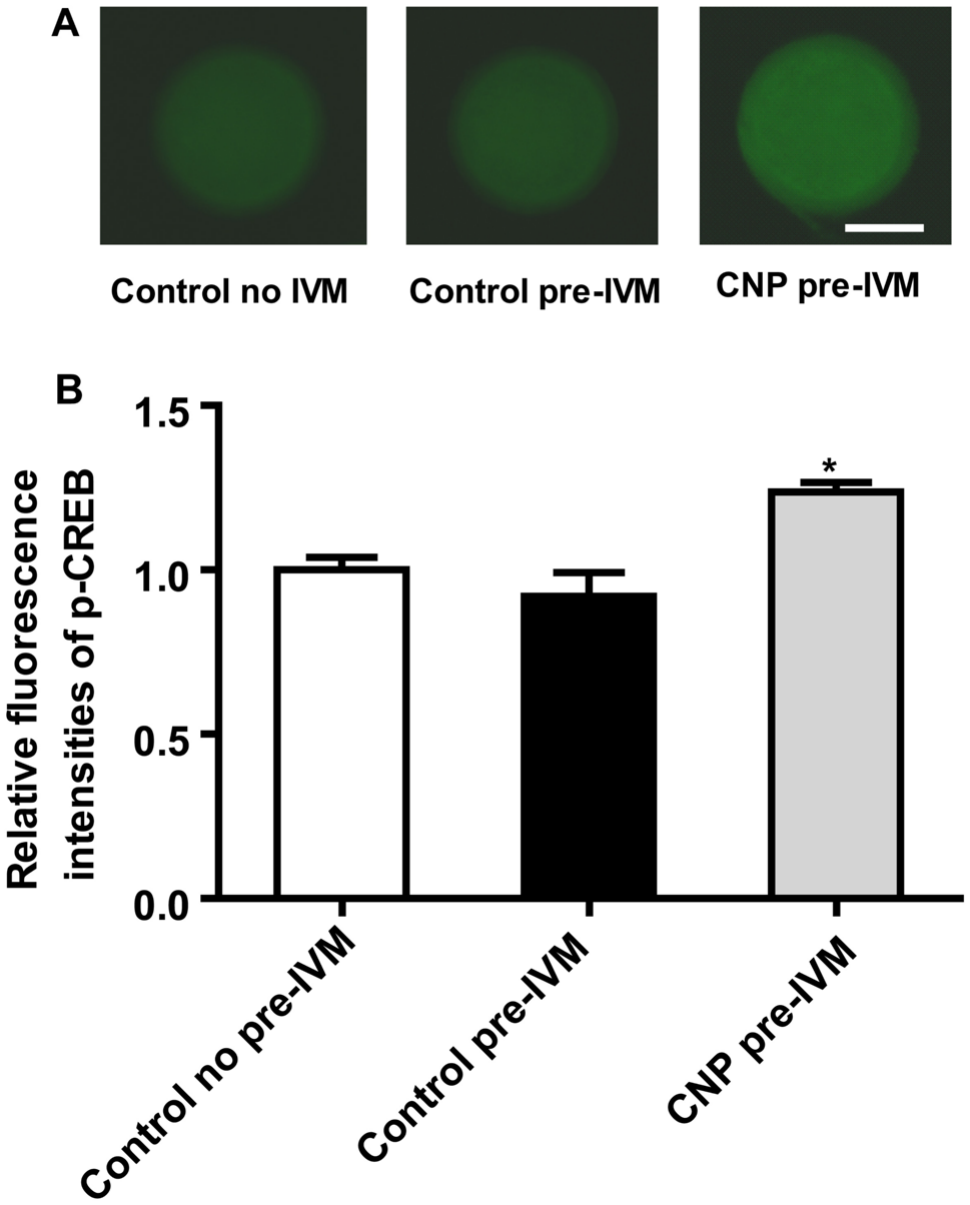
Effects of CNP treatment before IVM on abundance of p-CREB protein during bovine oocytes maturation. Immature cumulus-oocyte complexes (COCs) were subjected to IVM for 24 h (Control no pre-IVM), or cultured in pre-IVM medium supplemented without or with CNP (Control pre-IVM and CNP pre-IVM, respectively) for 6 h, followed by 24 h of IVM. (**A**) Representative immunofluorescence images of the p-CREB in bovine oocytes from each treatment group. Scale bar = 50 µm (**B**) Quantitative analysis of abundance of bovine oocytes p-CREB protein by immunostaining in each treatment group after IVM. Compared with the oocytes in the control (no pre-IVM) and pre-IVM (no CNP) group, *P < 0.05. The data are from at least three independent experiments.

To assess the effect of CNP pre-IVM treatment on CREB activity in bovine oocytes, collected immature oocytes were either directly in vitro matured for 24 h (Control), or cultured in basic pre-IVM medium supplemented without or with CNP (Control pre-IVM and CNP pre-IVM, respectively) for 6 h, followed by IVM for 24 h. After IVM, the protein levels of p-CREB in oocytes of each treatment group were detected. The results showed that the protein levels of p-CREB in CNP pre-IVM treatment group was significantly higher than that in the control (no pre-IVM) and pre-IVM (no CNP) group at the end of IVM (*P* < 0.05; Fig. 2).

### CNP treatment before IVM increases the protein levels of p-CREB in oocytes matured in vitro by acting on NPR2

**Figure 3:**
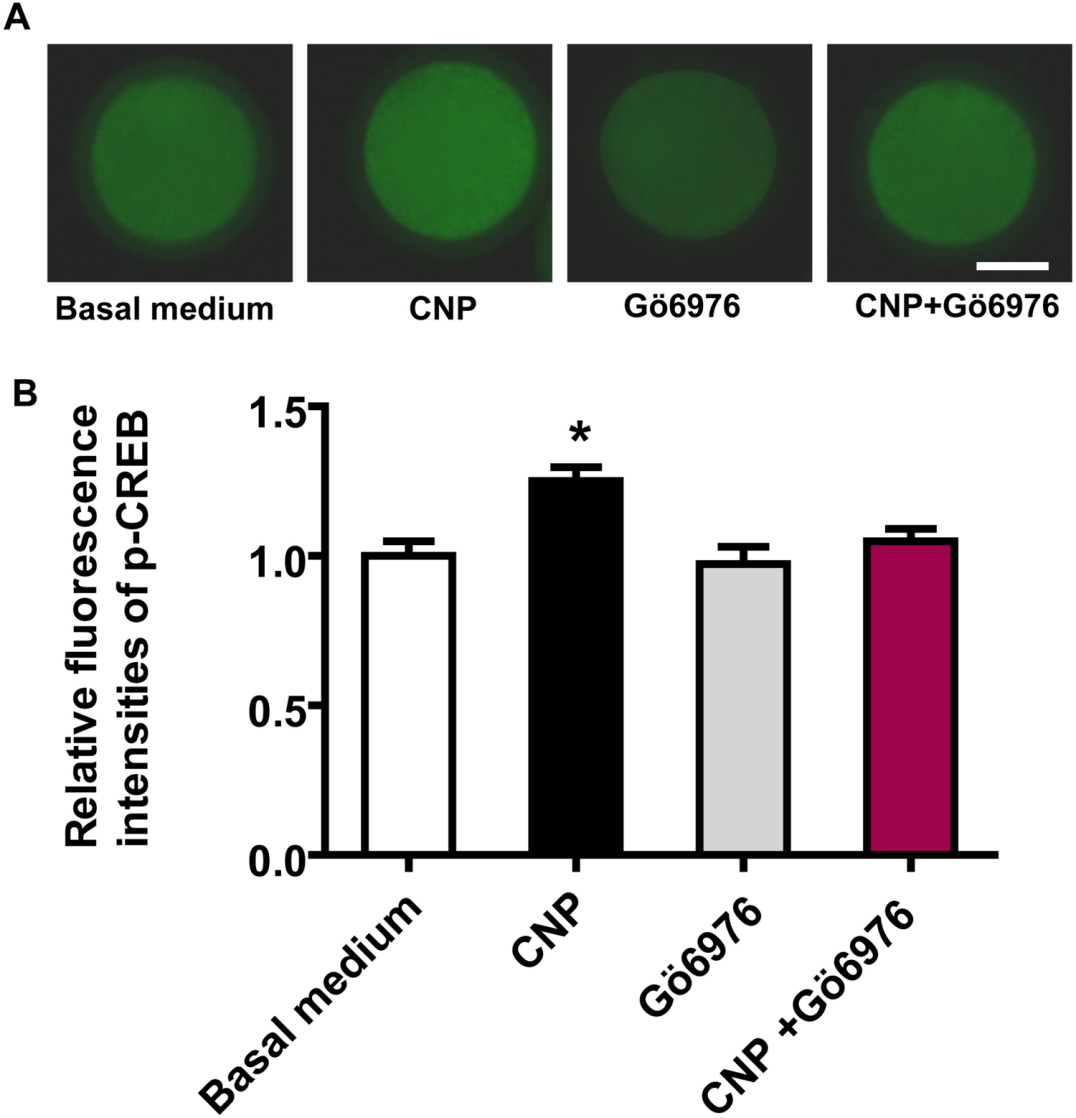
NPR2 inhibition abrogates CNP-stimulated CREB phosphorylation during bovine oocytes maturation. Immature cumulus-oocyte complexes (COCs) were treated in basal medium in the absence or presence of 200 nM CNP and/or 5 μM Gö6976, a competitive inhibitor of NPR2 (Basal medium, CNP, Gö6976, and CNP + Gö6976, respectively) for 6 h, followed by IVM for 24 h. (**A**) Representative immunofluorescence images of the p-CREB in bovine oocytes from each treatment group. Scale bar = 50 µm. (**B**) Quantitative analysis of abundance of bovine oocytes p-CREB protein by immunostaining in each treatment group after IVM. *P < 0.05. The data are from at least three independent experiments.

It is well known that CREB can be activated by cAMP or cGMP. In addition, CNP has been showed to maintain higher levels of cGMP and cAMP in bovine oocyte via activating NPR2. This led us to hypothesize that CNP pre-IVM treatment activates bovine oocytes CREB by acting on NPR2. To test this possibility, collected immature COCs were treated in basal medium in the absence or presence of 200 nM CNP and/or 5 μM Gö6976, a competitive inhibitor of NPR2^4^ (Basal medium, CNP, Gö6976, and CNP + Gö6976, respectively) for 6 h, followed by IVM for 24 h. After IVM, the protein levels of p-CREB in oocytes of each treatment group were examined. As shown in Fig. 3, compared to basal medium group, the elevated the protein levels of p-CREB in oocytes was no longer observed in the CNP + Gö6976 group. Furthermore, compared to CNP treatment alone, the protein levels of p-CREB in oocyte was significantly decreased (*P* < 0.05) when COCs were treated by CNP and Gö6976 simultaneously (Fig. 3).

### CNP treatment before IVM modulates mitochondria biogenesis in oocyte matured in vitro by acting on CREB.

**Figure 4:**
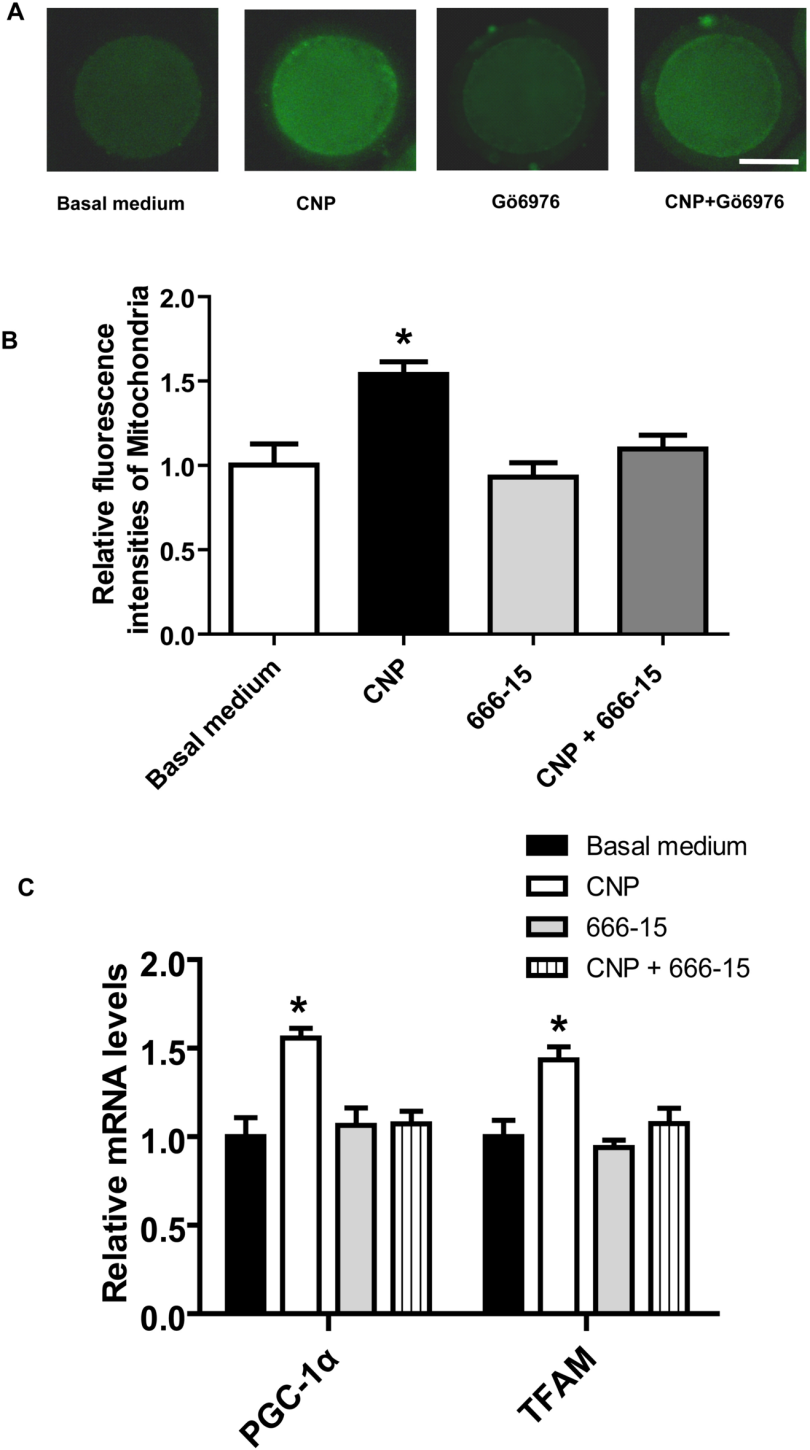
Inhibition of CREB abolishes the beneficial effect of CNP treatment before IVM on mitochondria biogenesis during bovine oocytes maturation. Immature cumulus-oocyte complexes (COCs) were treated in basal medium in the absence or presence of 200 nM CNP and/or 1 μM 666-15, a specific CREB inhibitor (Basal medium, CNP, 666-15 and CNP+666-15, respectively) for 6 h, followed by 24 h of IVM. (**A**) Representative images of the oocyte stained with MitoTracker green in each treatment group after IVM. Scale bar = 50 µm. (**B**) Relative fluorescence intensities of the mitochondrial content in each treatment group oocytes after IVM. (**C**) Relative mRNA transcript abundance of genes modulating mitochondria biogenesis in bovine oocytes from each treatment group after IVM. *P < 0.05. The data are from at least three independent experiments.

As shown in Fig. 2, CNP promoted the expression of p-CREB, which may be associated with enhanced mitochondria biogenesis in bovine oocytes. Hence, we hypothesized that CNP enhances mitochondria biogenesis in oocytes by acting on CREB. To test this possibility, collected immature oocytes were treated in basal medium in the absence or presence of 200 nM CNP and/or 1 µM 666-15, a specific CREB inhibitor^17^ (Basal medium, CNP, 666-15, and CNP+666-15, respectively) for 6 h, followed by IVM for 24 h. After IVM, the intensity of the mitochondria and gene expression of key regulators related to mitochondrial biogenesis in oocytes of each treatment group were detected. As shown in Fig. 4, compared to basal medium group, the increased level of mitochondria content and upregulated expression of genes related to mitochondrial biogenesis in oocyte was no longer observed in CNP+666-15 group. Furthermore, compared to CNP treatment alone, the mitochondria content and expression levels of genes related to mitochondrial biogenesis in oocyte was significantly decreased (*P* < 0.05) when COCs were treated by CNP and 666-15 simultaneously (Fig. 4).

## Discussion

It is well established that mitochondria are the most abundant organelle located in the cytoplasm and is often regarded as an important marker of oocyte quality. Thus, we explored the effect of CNP pre-IVM on bovine oocytes mitochondrial biogenesis in vitro and attempted to elucidate the related mechanism. Our findings demonstrated that pre-IVM treatment of bovine oocyte with CNP increased mitochondria content. Moreover, a previous study found that mtDNA copy number had been increased during bovine oocytes maturated in vitro following CNP pre-IVM treatment^4^. These findings indicated that CNP pre-IVM treatment may promoted the mitochondrial biogenesis in bovine oocytes. Besides, previous studies have suggested that the modulation of mitochondrial biogenesis is associated with enhanced cytoplasmic maturation in porcine oocytes^18^, indicating that CNP pre-IVM may contribute to enhanced cytoplasmic maturation in bovine oocytes by regulating mitochondria biogenesis. However, the underlying molecular mechanism on CNP-induced mitochondria biogenesis in bovine oocyte largely remain unknown.

Mitochondrial biogenesis is an important biological process for maintaining normal mitochondrial homeostasis, which involves replication of mitochondria DNA (mtDNA) and expression of nuclear and mitochondrial genes^19^. PGC-1α is known to be a central transcriptional coactivator that induces mitochondrial biogenesis^20^. It is well known that activation of PGC-1α induces the expression of NRF1/NRF2, leading to transcription of nuclear-encoded mitochondrial proteins and of TFAM. TFAM activates transcription and replication of the mitochondrial genome^21^^.^ In addition, numerous studies have shown that the increased expression of PGC-1α enhanced mitochondrial biogenesis by modulating the gene expression of NRF1, NRF2 and TFAM, resulting in the upregulation of mitochondrial electron transport chain proteins encoded by both nuclear and mitochondrial genomes^10,11,20^. In this study, PGC-1α expressions was markedly upregulated in the CNP pre-IVM oocytes than that in control (no pre-IVM) and pre-IVM (no CNP) oocytes. In addition, the mRNA expression of PGC-1α downstream genes, including transcription factors responsible for stimulating mitochondrial biogenesis (NRF1, NRF2 and TFAM) and proteins of the mitochondrial electron transport chain encoded by both nuclear and mitochondrial genomes was also significantly increased following CNP pre-IVM treatment, indicating that CNP participates in the modulation of mitochondria biogenesis via the upregulation of PGC-1α.

CREB is known to be an important activator of PGC-1α transcription by binding to the PGC-1α promoter^22^. In addition, CREB activity is known to be regulated by phosphorylation^23^. It is well established that cyclic adenosine 3′,5′-monophosphate (cAMP) and cyclic guanosine 3′,5′-monophosphate (cGMP), are key cyclic nucleotides controlling mammalian oocyte maturation^24^. Moreover, previous studies had demonstrated that higher levels of cGMP /cAMP in animal somatic cells can induced CREB phosphorylation by activating protein kinase (PKG) or protein kinase (PKA)^25,26^. It has been widely accepted that CNP can maintain oocyte meiotic arrest by inducing higher levels of cGMP and cAMP in animal oocyte via activation of NPR2 located in cumulus cells, implying its vital role in activating CREB. More importantly, there are evidence suggesting that activation of CREB using different bioactive compounds can induce mitochondria biogenesis in a variety of cell types^27–30^. Therefore, we hypothesized that CNP may induce bovine oocyte mitochondria biogenesis by activating CREB.

To further explored the possible molecular mechanism underlying CNP-induced mitochondrial biogenesis in bovine oocyte. We first investigated whether CNP can induce CREB phosphorylation. As expected, the results showed that CNP pre-IVM treatment increased the phosphorylation of CREB in bovine oocytes after IVM. However, it has been identified that inhibition of NPR2 activity using Gö6976, a relatively specific inhibitor, can effectively abrogates CNP-stimulated CREB phosphorylation in bovine oocytes. In addition, we previously found that inhibition of NPR2 activity using Gö6976, prevented CNP-induced elevation in cGMP and cAMP levels in bovine oocyte^31^. These results revealed that CNP can activate CREB via the maintenance of higher levels of cGMP/cAMP in bovine oocyte, which is similar to those foundation in animal somatic cells by modulating effects of cGMP/cAMP on CREB activity^25,26^. Furthermore, in this study, we further found that inhibition of CREB function by 666-15, a specific inhibitor, completely abolished the positive effects of CNP on the mRNA expression of PGC-1α and TFAM and mitochondrial content in bovine oocytes. Since CREB has been demonstrated to be the upstream regulatory signal of PGC-1α^32,33^. Therefore, our data indicated that CNP is a positive activator of CREB and PGC-1α induction in bovine oocytes. CREB signal pathway is one of importantly regulatory mechanism in bovine oocytes maturation, and its activation exerts a positive effect on bovine oocytes mitochondrial biogenesis. Taken together, these findings support the view that CNP induces mitochondrial biogenesis in bovine oocyte via activation of CREB.

## Materials and Methods

Our research was approved by the Laboratory Animal Resource Center of Inner Mongolia Minzu University (Approval No. 2022-0609) and was performed in accordance with the Animal Care and Use Statute of China.

### Chemicals

All chemicals used in this study were purchased from Sigma-Aldrich, a company based in St. Louis, USA, except for those that are specifically defined. Hepes-buffered tissue culture medium 199 (HTCM199, 12,340-030), TCM199 (11,150-059) were purchased from GIBCO (Grand Island, NY, USA).

### COCs

Bovine ovaries were collected from a local abattoir and transported to the laboratory within 2 h in 0.9% saline containing antibiotics at 28–30 °C. Follicular fluid was aspirated from 3 to 6 mm antral follicles using a syringe needle. Only cumulus-oocyte complexes (COCs) with a homogeneous cytoplasm and multiple compact cumulus cell layers were collected under a stereomicroscope. After that, COCs were cleaned in HEPES-buffered TCM199 medium containing with 0.1% (w / v) polyvinyl alcohol (PVA).

### Pre in vitro maturation (pre-IVM)

COCs were cultured in 50-μL drops (~ 10 COCs) of pre-IVM medium covered with mineral oil for 6 h at 38.5 °C under 5% CO_2_ in humidified air. The pre-IVM medium used was modulated according to the experimental design as (1) Basic pre-IVM medium: TCM199 with Earle’s salts supplemented with 0.4% fatty acid-free BSA). (2) CNP supplementation: Basic pre-IVM medium supplemented with CNP (200 nM). (3) Effect of inhibitors: The inhibitors of CREB or NPR2 were added in basic pre-IVM medium or CNP-supplemented pre-IVM medium.

### IVM

COCs were cultured in 50-ul drops (~ 10 COCs) of IVM medium covered with mineral oil for 24 h at 38.5 °C under 5% CO2 in humidified air. IVM medium was adapted from Zhenwei et al.^5^ TCM199 supplemented with 10% FBS, 10 µg/ml FSH, 1 µg/ml LH and 1 µg/ml estradiol.

### Mitochondrial staining

Mitochondrial content were evaluated with MitoTracker green (Molecular Probes, Eugene, OR, USA). Denuded oocytes were incubated with 100 nM MitoTracker green in TCM199-based medium for 30 min at 37 °C in the dark, washed three times in PBS, and then fixed with 4% paraformaldehyde-PBS for 15 min at 37 °C. After fixation, oocytes were washed three times in PBS, and mounted and examined under a fluorescence microscope (Eclipse Ci-s, Nikon, Tokyo, Japan). Image J software (National Institutes of Health, Bethesda, MD, USA) was used to analyze fluorescence intensity.

### Quantitative real time PCR analysis

A CellAmp Direct SYBR RT-qPCR Kit (Cat #3735S, Takara Bio Inc., Shiga, Japan) was used to produce cDNA from denuded oocyte RNA according to the manufacturer’s instructions. Briefly, 50 denuded oocytes of each group were lysed in Cell Lysis solution composed of 48 μL Cell Lysis II Buffer and 2 μL DNase I at room temperature for 5 min. Reverse transcription was performed using a one‐step procedure. Briefly, 2 μL cell lysate, 4 μL 5 × CellAmp Buffer II, 1μL PrimeScript RT Enzyme Mix, 1 μL RT Primer Mix and 12 μL RNase Free H_2_O were mixed in a nuclease-free microfuge tube, and then incubated at 37 °C for 30 min, and at 85 °C for 5 s. Complementary DNA was stored at − 20 °C until used. Quantification of cDNA was then performed using an ABI-Stepone Plus instrument (Applied Biosystems, Carlsbad, CA, USA) with SYBR Green reaction mix (Cat #3735S, Takara Bio Inc., Shiga, Japan). Each PCR was performed with an initial denaturation step at 95 °C for 30 s followed by 40 cycles consisting of 5 s of denaturation at 95 °C and 15 s of annealing/extension at 60 °C. *GAPDH* was used as a reference gene. Relative gene expression was calculated by the 2^−ΔΔCt^ method. The primers used are listed in Table 1. Each experiment was repeated independently three times.

**Table 1:** Primer sequences used for real-time PCR.

Gene	Primer	GenBank Accession No
PGC1α	GGCAATTGTCAGGTTGGAGT	XM_059887363.1
	AAAAGTCACGTCGGCCATAC	
NRF1	GAGGCAGGGGTGGGCAATAA	NM_001098002.2
	TGGGAATAGGGTGGGTGAGG	
NRF2	CACATCACGACCATCTCAG	XM_010800937.4
	CCTTCATTACCCAAACCAC	
TFAM	GCCGATTTCCCATAGTGC	NM_001034016.2
	ATCCGCTCCTGACTTTCC	
NDUFV1	GGTACAAGACGAAGGAGATT	XM_010821048.3
	GCGTTCACCACCAGATAC	
NDUFS2	AATGGGCAGAGCAGTACG	XM_010802740.4
	AAAGTTCAGGGTCAGGTTC	
CYC1	CAATGAAGATGGGGAGATG	NM_001038090.2
	CCAGGAAAATAAGGGTTGAAGT	
COX5A	TCACTTCGCTGCTACTCC	XM_005221914.3
	GTGTTCATCCCTTTACGC	
ND1	CTCCGAAAAGGTCCAAATGT	AY526085.1
	AGATGTAGCGGGTCGTAGTG	
ND2	CCCACGAGCTACAGAAGCAT	AY526085.1
	TAGGGGGATGCCCTGTGTTA	
CYTB	TTACGGGTCTTACACTTTTC	AY526085.1
	ATCCGCCTCAGATTCATT	
GAPDH	AGATAGCCGTAACTTCTGTGC	
	TGGGTGGAATCATACTGGAAC	NM_001034034.2

### Immunofluorescence detection

The effects of CNP pre-treatment on the levels of p-CREB in oocytes were examined using immunofluorescence. In brief, denuded oocytes fixation was performed using 4% paraformaldehyde-PBS containing 0.1% (w/v) PVA for 30 min and then in 0.5% Triton-X 100-PBS permeabilized for 30 min, all at room temperature. The primary and secondary antibodies used for this procedure were rabbit polyclonal anti-p-CREB (1:500; ab254107, abcam, Cambridge, UK) and fluorescein-conjugated goat anti-rabbit IgG (1:100; 4412S, Cell Signaling Technology, Danvers, MA, USA), respectively. The stained oocytes were mounted on glass sides and examined under fluorescence microscope (Eclipse Ci-s; Nikon). The settings of imaging and analysis are same as all experiments. The fluorescence levels were analyzed using ImageJ software.

### Statistical analysis

All statistical tests were performed using SPSS software version 18.0 (IBM, USA). All experiments were repeated at least three times and data are expressed as means ± SEM. All data obtained in the present study were assessed by one-way ANOVA, followed by Tukey's test. Values were considered significantly different at *P* < 0.05.

## Data Availability

All the data supporting the findings of this study are contained within the paper.
